# Deep learning for quality assessment of axial T2-weighted prostate MRI: a tool to reduce unnecessary rescanning

**DOI:** 10.1186/s41747-025-00584-z

**Published:** 2025-04-29

**Authors:** Jacob N. Gloe, Eric A. Borisch, Adam T. Froemming, Akira Kawashima, Jordan D. LeGout, Hirotsugu Nakai, Naoki Takahashi, Stephen J. Riederer

**Affiliations:** 1 Department of Radiology, Mayo Clinic, Rochester, MN USA; 2 Department of Radiology, Mayo Clinic, Phoenix, AZ USA; 3 Department of Radiology, Mayo Clinic, Jacksonville, FL USA; 4 Kyoto University, Kyoto, Japan

**Keywords:** Artificial intelligence, Deep learning, Magnetic resonance imaging, Prostate, Time management

## Abstract

**Background:**

T2-weighted images are a critical component of prostate magnetic resonance imaging (MRI), and it would be useful to automatically assess image quality (IQ) on a patient-specific basis without radiologist oversight.

**Methods:**

This retrospective study comprised 1,412 axial T2-weighted prostate scans. Four experienced uroradiologists graded IQ using a 0-to-3 scale (0 = uninterpretable; 1 = marginally interpretable; 2 = adequately diagnostic; 3 = more than adequately diagnostic), binarized into nondiagnostic (IQ0 or IQ1), requiring rescanning, and diagnostic (IQ2 or IQ3), not requiring rescanning. The deep learning (DL) model was trained on 1,006 scans; 203 other scans were used for validation of multiple convolutional neural networks; the remaining 203 exams were used as a test set. 3D-DenseNet_169 was chosen among 11 models based on multiple evaluation criteria. The rescan predictions were compared to the number of rescans performed on a subset of 174 exams.

**Results:**

The model accurately predicts radiologist IQ scores (Cohen *κ* = 0.658), similar to the human inter-rater reliability (*κ* = 0.688–0.791). The model also predicts rescanning necessity similarly to radiologists: model *κ* = 0.537; reviewer *κ* = 0.577–0.703. The rescan model prediction area under the curve was 0.867.

**Conclusion:**

The DL model showed a strong ability to differentiate diagnostic from nondiagnostic axial T2-weighted prostate images, accurately mimicking expert radiologists’ IQ scores. Using the model, the clinical unnecessary rescan rate could be reduced from over 50% to less than 30%.

**Relevance statement:**

DL assessment of T2-weighted prostate MRI scans can accurately assess IQ, determining the need to repeat inadequate scans as well as avoiding repeat scans of those with adequate diagnostic quality, resulting in reduced unnecessary rescanning.

**Key Points:**

- Artificial intelligence assessment of prostate MRI T2-weighted image quality can improve exam time management.
- The model showed over 75% accuracy in assessing prostate MRI T2-weighted image quality.
- Expert radiologists have a substantial agreement in evaluating prostate MRI T2-weighted image quality.

**Graphical Abstract:**

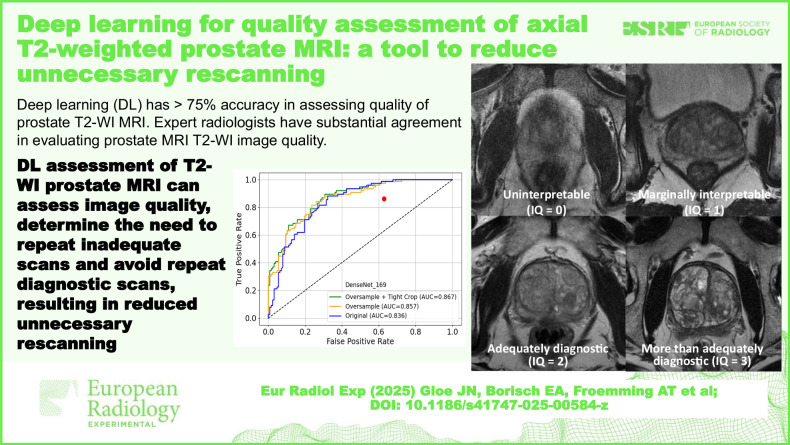

## Background

The current standard for imaging men suspected of prostate cancer is multiparametric magnetic resonance imaging (mpMRI), comprised of T2-weighted imaging (T2WI), diffusion-weighted imaging (DWI), and dynamic contrast-enhanced imaging sequences [1, 2]. The Prostate Imaging-Reporting and Data System (PI-RADS) version 2.1 [3], specifies acquisition parameters for the underlying sequences. However, adhering to these guidelines does not guarantee quality, as factors such as motion or metal implants can adversely affect quality [4–8].

Poor image quality (IQ) in T2WI and DWI has been shown to adversely affect prostate cancer detection rates [9, 10]. In 2020 the Prostate Imaging Quality (PI-QUAL) system was created to standardize IQ in mpMRI [11]. PI-QUAL assesses a mpMRI exam based on PI-RADS technical parameters and a qualitative scale for diagnostic quality [12]. It has recently been updated to version 2 [13], in which a total of ten criteria are assessed in determining a score. This version also recommends that poor-quality sequences should be repeated while the patient is undergoing the examination.

In parallel with the developments in PI-RADS and PI-QUAL, there has been the use of deep learning (DL) in prostate MRI. Applications have included the detection and classification of prostate cancer [14–17], segmentation of the prostate [18, 19], and denoising of T2WI [20–22]. DL has also been utilized to perform IQ assessments such as evaluation of the T2WI component of a prostate mpMRI exam [23, 24] or of the T2WI and DWI components of biparametric MRI [25]. These results have been effective in showing the viability of DL for assessing IQ in prostate MRI, although they do not provide a quantitative measurement of the benefits this type of assessment could have clinically.

If an MRI scan is determined to have nondiagnostic quality, it is desirable to compensate, if possible, such as by repeating the original scan. For a T2WI scan deemed corrupted by motion, another option is to use a motion-resistant T2WI sequence, such as PROPELLER [26]. Such sequences can reduce motion artifacts in T2WI prostate MRI [27, 28], but acquisition times are typically 50% longer than conventional Cartesian T2WI. In both cases, the choice of assigning diagnostic or nondiagnostic quality to an initial T2WI scan can impact the duration of the exam. A DL algorithm may aid in accurately making this assignment.

The purpose of this work was to develop a DL model for assessing the IQ of T2WI prostate MRI scans for motion corruption and further illustrate its potential usage in determining the necessity of rescans.

## Methods

### Ethics, patients’ selection, and MRI protocol

This retrospective single-institution three-site study was performed under IRB protocol 23-008038 in which the requirement for patient consent for participation was waived at all three sites.

**Fig. 1:**
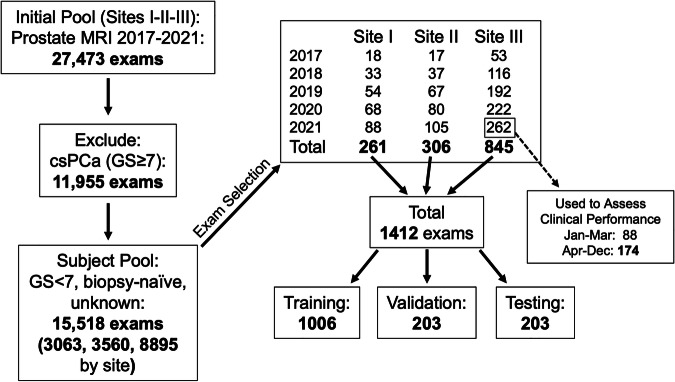
Flow in the selection of the 1,412 exams from the three sites

Data were taken from clinically indicated prostate MRI exams from three sites collected from 2017 to 2021, an initial total of 27,473 exams (Fig. 1). To limit the model to prostate MRI exams of men suspected of but without known clinically significant prostate cancer, exams from subjects with Gleason Score (GS) ≥ 7 were excluded, resulting in 15,518 exams from patients having various pre-MRI statuses, including biopsy-naïve, biopsy-negative, and unknown. Exams were then selected in approximate proportion to the number of prostate MRI exams performed at each site.

In accumulating a dataset for any DL IQ model, it is desirable to have sufficient representation across the range of IQ scores. Because poor-quality exams are generally acquired less frequently than medium- or high-quality exams, an attempt was made at the outset to preferentially include poor-quality exams. Exams were initially selected by considering the written quality section of the MRI report. If the report contained the keywords ‘T2’ and ‘motion’, the exam was considered likely to be of poor quality, and these exams were selected before others, resulting in 1,175 exams. To increase this number, four preliminary DL models on IQ were utilized to evaluate a second pass of 1,200 exams, and those deemed poor quality by at least one model (237 exams total) were added to the dataset. This resulted in the final dataset of 1,412 exams shown in Fig. 1.

For all exams, the T2WI series had been acquired axially through the prostate using a multislice fast or turbo spin-echo acquisition. Of the 1,412 exams, 1,408 (99.7%) used slice thickness ≤ 3 mm, *i.e*., they were compatible with the PI-QUALv2 T2WI acquisition criterion. In all cases, the repetition time was ≥ 3,000 ms, and the echo time was ≥ 95 ms. The median number of slices was 36 (interquartile range 32–39). The median patient age was 68 years (interquartile range 61–72). Variability in the in-plane field of view and sampling resolution is shown in Table 1, variability in scanner platforms is in Supplemental Table S1. Of the 1,412 exams, 146 (10.3%) were performed using an endorectal coil; 45 (3.2%) at 1.5 T, and the remaining 1,367 (96.8%) at 3 T.

**Table 1:** Description of the resolutions and fields of view for the 1,412 T2WI prostate scans

LR × AP in-plane sampling	LR × AP field of view (mm^2^)	LR × AP acquired in-plane resolution (mm^2^)	Counts
230 × 400	160 × 160	0.70 × 0.40	551
320 × 320	180 × 180	0.56 × 0.56	252
320 × 320	200 × 200	0.63 × 0.63	206
320 × 320	240 × 240	0.75 × 0.75	111
256 × 320	160 × 160	0.63 × 0.50	70
224 × 384	200 × 200	0.89 × 0.52	49
368 × 313	180 × 180	0.49 × 0.58	25
288 × 320	180 × 180	0.63 × 0.56	25
224 × 416	180 × 180	0.80 × 0.43	19
256 × 320	180 × 180	0.70 × 0.56	17
Other	Other	Other	87

Left-right (*LR*) and anterior-posterior (*AP*) correspond to the phase encoding and frequency encoding directions, respectively

### Quality assessment by expert radiologists

Four experienced uroradiologists (A.F., A.K., J.L., N.T.), each of them with over 7 years of experience in prostate MRI, representing the three sites, reviewed the T2WI scans on workstations without knowledge of clinical information. Each reviewer provided blinded ratings of IQ using a four-point scale (0 = uninterpretable; 1 = marginally interpretable; 2 = adequately diagnostic; 3 more than adequately diagnostic) by subjectively assessing the level of noise, the ability to delineate structures over the entire prostate gland, and the degree of artifact such as blurring and stepladder artifact, defined as any slice-to-slice displacement. The IQ scores were subsequently also grouped into a two-point scale: nondiagnostic (IQ scores of 0 or 1), requiring a rescan or diagnostic (IQ scores of 2 or 3), not requiring a rescan. Prior to the individual reviews, 16 exams from the 1,412 (four for each IQ category) were assessed by the four radiologists and agreed upon in consensus, serving as references for subsequent independent scoring (Supplemental Video S1). For the review, the scans were cropped to a 12-cm field of view, and the central 16 axial images encompassing the central gland were evaluated.

Of the 1,412 exams, 1,209 were divided evenly among the four reviewers to evaluate individually. To assess inter-reader scoring consistency, 65 exams were scored by all four reviewers, and 138 exams were scored by exactly two reviewers. For the 203 exams with more than one reviewer, the median IQ score was used as the true label, with one reviewer (NT) acting as arbitrator to ensure integer values.

Inter-reader consistency was assessed using quadratic-weighted Cohen *κ* [29] and Krippendorf *α* with interval weighting [30].

### DL model design

The 1,412 exams were apportioned as follows: the 203 exams having multiple reviewer scores were used as the test set. The remaining 1,209 exams were split into 1,006 for training and 203 for validation, stratified according to the IQ score percentages for similar score distributions.

Eleven three-dimensional convolutional neural network architectures were considered to predict IQ scores (Supplemental Table S2). All models were implemented using the MONAI framework [31] and coded using PyTorch v2.0.1 [32].

Before training, to limit memory, several preprocessing steps were done. First, only 16 central axial slices of each exam were used. Second, because the prostate typically fills only a small portion of the in-plane field of view, a tight crop around the prostate was implemented using an in-house segmentation model to crop each slice according to the largest prostate area within the volume. After cropping, Lanczos interpolation [33] was used to provide uniform within-slice sampling (256 × 256 pixels) for input into the model, finer than the acquired in-plane resolution in all cases. Finally, pixel values were normalized according to the mean and standard deviation for each scan to standardize scan intensity.

Because the uninterpretable (IQ = 0) exams are underrepresented in the dataset (119 of the 1,412 exams, 8.4% of the total), these exams were oversampled using a bootstrapping method [34] in which randomly selected copies of IQ = 0 exams are added to the training set with replacement until there were as many as the majority class (IQ = 2, 578 exams total). This artificially inflated the number of samples in the minority class with the aim of increasing the performance of the model in that class. An exponential learning rate scheduler with *γ* = 0.99 was implemented to help alleviate overfitting, as well as left-right reflection, translation, and rotation data augmentations [35].

Hyperparameters for the chosen model were fine-tuned using the following values: the optimizer used was Adam [36] with *ε* = 1e-8 and a weight decay of 1e-2, and the loss function used was mean square error (MSE), preserving the ordinal nature of the scores. The output of the chosen model was rounded to the nearest integer and truncated to lie in the range 0-to-3 to allow comparison with the radiologist-assigned IQ scores. Early stopping was utilized to halt training if the validation loss did not improve after 12 epochs.

### Model evaluation

The viability of the models to assess the quality of unseen MRI scans was determined using several different methods. Accuracies of both the IQ scores and the nondiagnostic or diagnostic rescan decision were calculated, defined as the percentage of cases in the test set in which the DL results exactly matched the radiologist’s assessment. Precision, also referred to as positive predictive value, measured how many scans predicted as nondiagnostic were actually nondiagnostic as determined via radiologist assessment. Recall, also referred to as sensitivity, measured the percentage of nondiagnostic scans correctly predicted as nondiagnostic by the model. The harmonic mean of precision and recall $\left(\frac{2* {Precision}* {Recall}}{{Precision}+{Recall}}\right)$, defined as the F1 score [37], was determined. Also reported was the Index Balanced Accuracy [38] as a summary of the accuracy of the model on each IQ category after accounting for any imbalance in the score distribution. Finally, before rounding and truncation, the model outputs were used for generating a receiver operating characteristic curve by varying the threshold for a rescan and comparing these results to the reviewer rescan decision.

To assess the consistency of each model, five-fold cross-validation was performed, each iteration with a unique training: validation (1006:203) split of the 1,209 cases not used for testing.

#### Comparison with clinical performance

The chosen model was compared to a reference clinical operating point for distinguishing nondiagnostic *versus* diagnostic scans determined as follows. At Site III when newly reconstructed T2WI images are observed to be motion-corrupted, the PROPELLER sequence is run to provide retrospective correction for in-plane motion. The decision to accept T2WI or rescan with PROPELLER would ideally be made by the radiologist overseeing the exam, but – due to practical issues—is left to the MRI technologist. Incorporation of PROPELLER into the mpMRI protocol was initiated at Site III in April 2021. Therefore, all exams from Site III included in the 845 total but acquired after March 31, 2021 (174 exams, Fig. 1) were reviewed to determine whether a PROPELLER scan was included. If so, an assignment of true positive or false positive was given, depending on whether the radiologist’s evaluation was nondiagnostic or diagnostic, respectively. Similarly, if a PROPELLER scan was absent, an assignment of true negative or false negative was given, depending on whether the radiologist’s determination was diagnostic or nondiagnostic. The tabulated results were then compared to the DL model results to determine any degree of improvement that automated quality assessment could provide relative to the clinical operating point.

## Results

### Expert radiologist score statistics

**Fig. 2:**
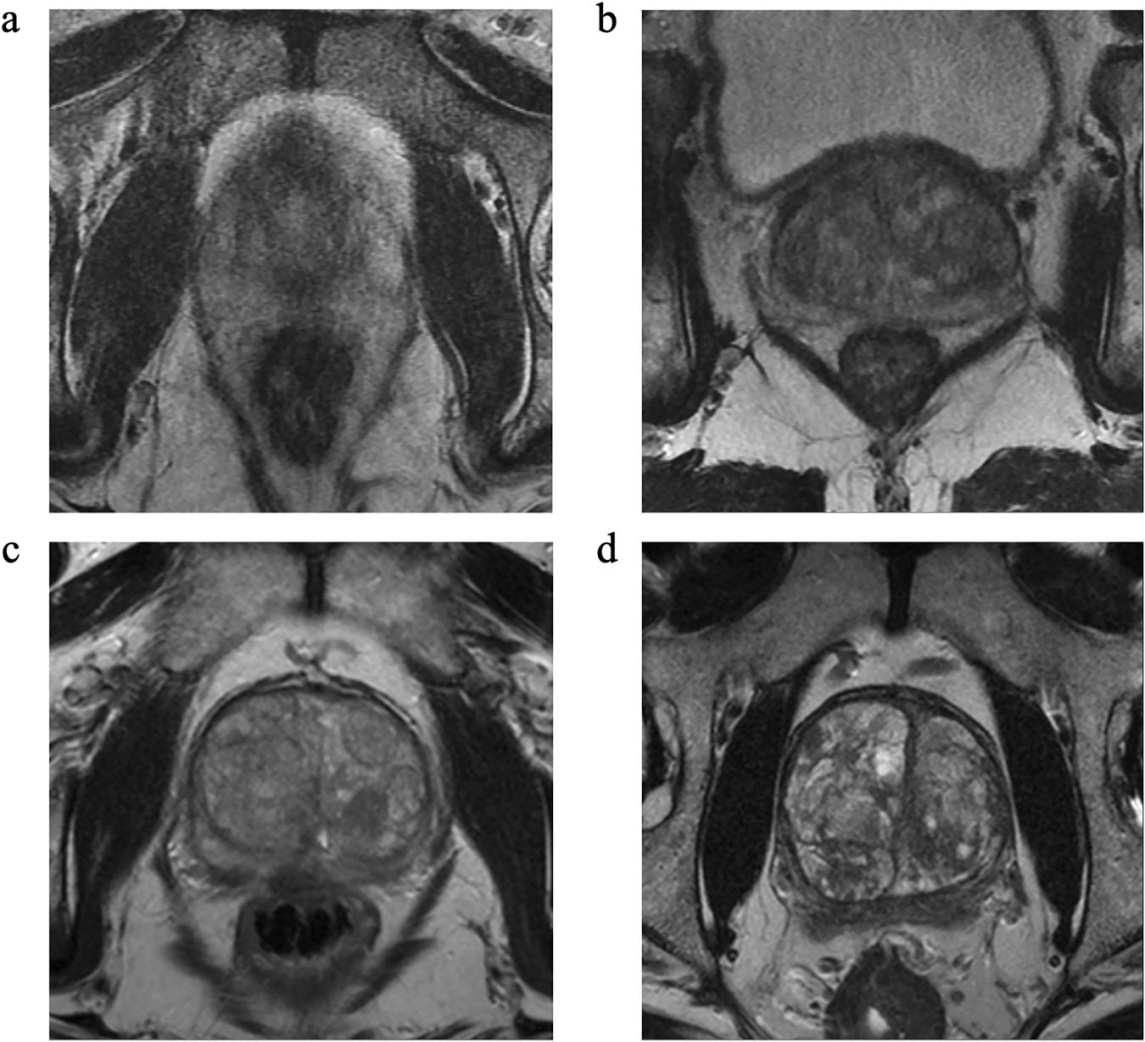
Examples of (**a**) uninterpretable (IQ = 0), (**b**) marginally interpretable (IQ = 1), (**c**) adequately diagnostic (IQ = 2), and (**d**) more than adequately diagnostic (IQ = 3) scans, as scored unanimously by the four expert radiologists. IQ, Image quality

**Fig. 3:**
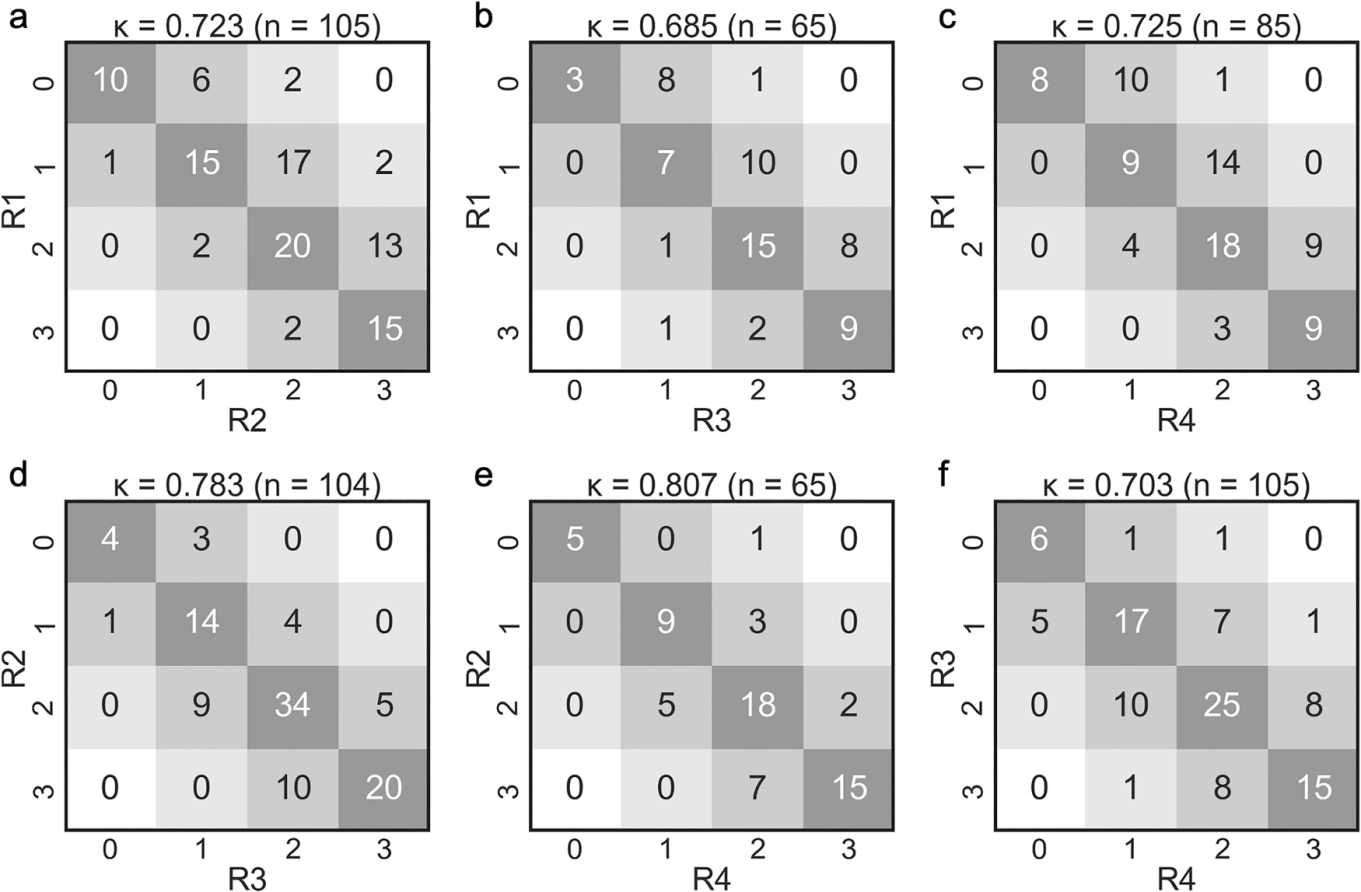
Inter-rater agreement matrices for the six pairs of four reviewers (R1–R4), including the Cohen *κ* score for each. Results are shown for (**a**) R1 *versus* R2, (**b**) R1 *versus* R3, (**c**) R1 *versus* R4, (**d**) R2 *versus* R3, (**e**) R2 *versus* R4, and (**f**) R3 *versus* R4.  Overall *κ* = 0.738 ± 0.043 (mean ± standard deviation). Krippendorf *α* = 0.73

**Fig. 4:**
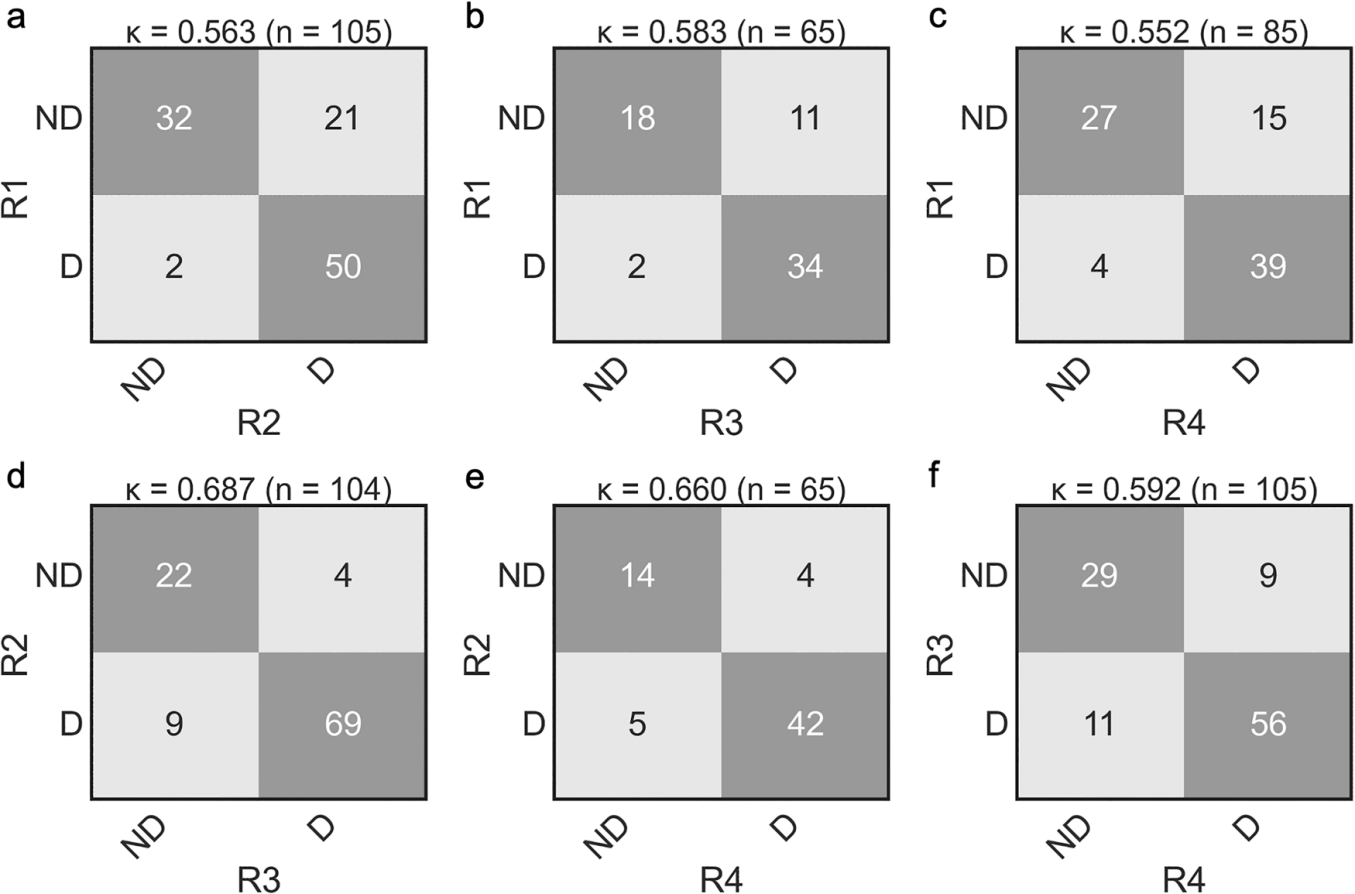
Inter-rater agreement matrices for rescan decisions for reviewers R1–R4 using nondiagnostic (ND) and diagnostic (D) labels, including the Cohen *κ* scores for each.  Results are shown for (**a**) R1 *versus* R2, (**b**) R1 *versus* R3, (**c**) R1 *versus* R4, (**d**) R2 *versus* R3, (**e**) R2 *versus* R4, and (**f**) R3 *versus* R4.  Overall *κ* = 0.606 ± 0.050 (mean ± standard deviation). Krippendorf *α* = 0.58

Radiologist scoring results from the 1,412 exams yielded the following score distribution: IQ = 119 exams (8.4%); IQ = 1,341 (24.1%); IQ = 2,578 (40.9%), and IQ = 3,374 (26.4%) (Supplemental Fig. S1). Figure 2 shows examples of IQ scores from 0 to 3. Figure 3 shows inter-reader agreement for the IQ scoring, the Cohen *κ* values (0.738 ± 0.043, mean ± standard deviation), and Krippendorf *α* (0.73), all indicating a substantial agreement [39]. Figure 4 shows analogous matrices for the nondiagnostic or diagnostic decision. The mean *κ* value (0.606 ± 0.050) shows a substantial agreement and *α* (0.58) a near-substantial agreement.

### DL model results

**Fig. 5:**
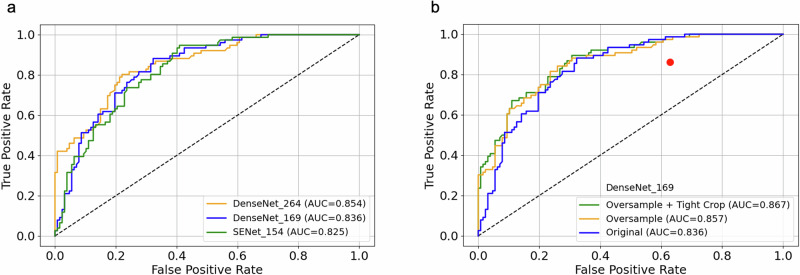
Receiver operating characteristic curve for rescan prediction, where the label ‘positive’ represents that the sequence is nondiagnostic and requires a rescan as determined by expert radiologist assessment (**a**) for the top three highest-performing models with a 12-cm field of view center crop and without minority oversampling, and (**b**) for the DenseNet_169 model, with and without oversampling and tight cropping around the prostate using the in-house segmentation model. The red dot is the reference clinical operating point (false-positive rate = 63%; true positive rate = 86%) for the 174 exams from Site III performed clinically from April 1, 2021 to December 31, 2021

Preliminary results for the eleven DL quality assessment models are shown in Supplemental Table S2. Figure 5a shows receiver operating characteristic curves for the three highest-performing models for the area under the receiver operating curve analysis (AUC) in classifying the test set exams as nondiagnostic and diagnostic. Based on its favorable scores as well as its consistency in cross-validation, DenseNet_169 (Supplemental Fig. S2) was chosen for further refinement [40]. Figure 5b shows that implementing oversampling of the minority class and tight cropping around the prostate slightly improve its AUC performance to 0.867.

**Fig. 6:**
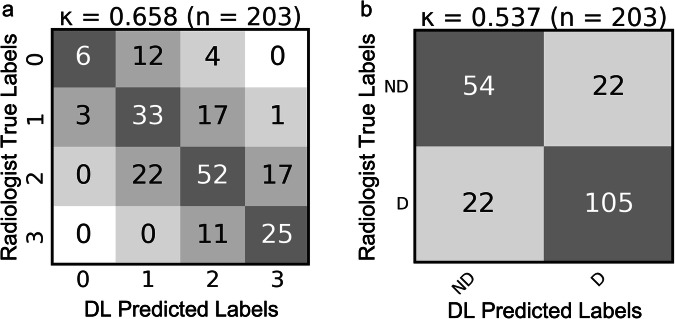
Agreement matrices comparing expert radiologist labels to deep learning-predicted labels for (**a**) image quality scores from 0 to 3, and (**b**) rescan decision using nondiagnostic (ND) and diagnostic (D) labels

On the test set, the model accurately predicts the IQ scores with an accuracy of 57.1% and a Cohen *κ* value of 0.658 (Fig. 6a), indicating substantial agreement. For rescan decision, accuracy is 78.3%, and the Cohen *κ* value drops to 0.537 (Fig. 6b), indicating a moderate to substantial ability of the model to differentiate between nondiagnostic and diagnostic scans. Furthermore, the F1 score for the rescan decision is 0.79. Index Balanced Accuracies for the four IQ labels are as follows: 0.25 for IQ = 0; 0.47 for IQ = 1; 0.40 for IQ = 2; and 0.59 for IQ = 3. This indicates that, after accounting for the imbalance in class distribution, the model performs best on the highest-quality scans. In making the decision for rescanning these values become more similar, 0.61 for diagnostic and 0.59 for nondiagnostic.

Results of the five-fold cross-validation for DenseNet_169 are shown in Table 2. Implementing minority oversampling and tight cropping slightly decreases average performance, but this is mostly due to the unusually high performance of the original DenseNet_169 model on the fifth fold (*K* = 5).

**Table 2:** Comparison of five-fold cross-validation evaluation metrics on the validation set for DenseNet_169 models

Metric	Model	*K* = 1	*K* = 2	*K* = 3	*K* = 4	*K* = 5	Average
IQ accuracy (%)	Original	55.1	55.6	51.9	53.1	61.7	55.5
	Oversample	51.9	52.2	48.1	54.7	57.6	52.9
	Oversample + Tight Crop	51.4	54.3	49.2	48.3	55.8	51.8
Rescan accuracy (%)	Original	79.8	83.1	82.3	82.3	84.3	82.4
	Oversample	77.8	79.8	79.0	80.7	80.7	79.6
	Oversample + Tight Crop	81.9	81.9	76.9	80.2	79.3	80.0
IQ Cohen *κ*	Original	0.60	0.58	0.62	0.57	0.71	0.62
	Oversample	0.58	0.58	0.60	0.66	0.65	0.61
	Oversample + Tight Crop	0.61	0.65	0.59	0.57	0.58	0.60

*IQ* Image quality, *K* Value of the fold

### Reference clinical operating point

**Fig. 7:**
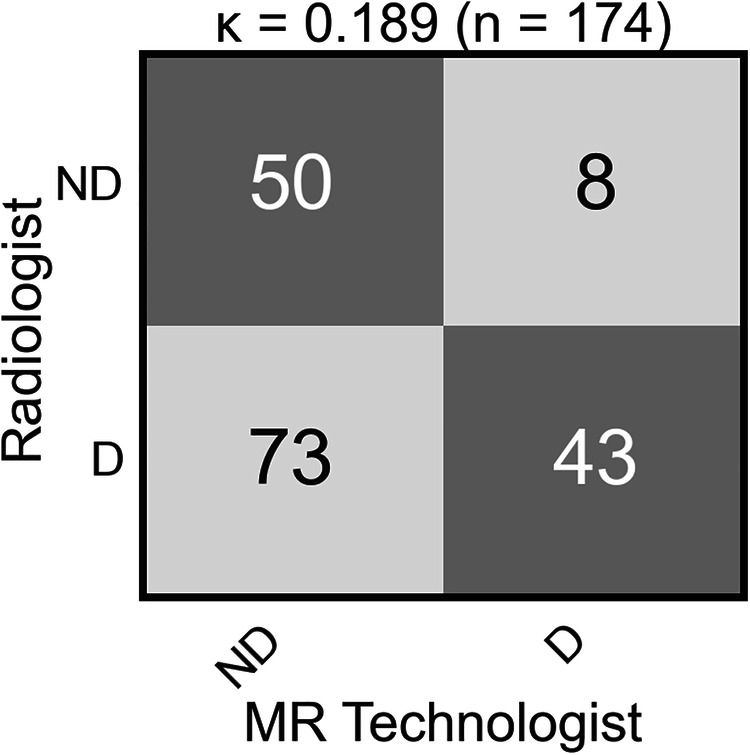
Expert radiologist *versus* technologist rescan decisions using nondiagnostic (ND) and diagnostic (D) labels on 174 scans from Site III acquired from April 1, 2021 to December 31, 2021

Figure 7 summarizes the technologist-assessed *versus* radiologist-based determination of nondiagnostic/diagnostic quality for the 174-exam subgroup performed at Site III to evaluate the clinical operating point. Fifty-eight exams were evaluated by the radiologists as nondiagnostic, and 50 of these agreed with the technologist’s determination, 8 did not, yielding a true positive rate of 86% (50/58). One-hundred sixteen exams were radiologist-designated as diagnostic and 43 of these were in agreement with the technologist determination, 73 did not, yielding a false-positive rate of 63% (73/116). The resultant operating point for clinical rescans was designated as the red point (Fig. 5b). Comparing this clinical operating point to the model performance, the unnecessary rescan rate in principle could be decreased from 63% to 28% while maintaining 86% sensitivity, potentially reducing unnecessary scan time. Supplemental Fig. S3 shows two examples of false-positive nondiagnostic exams.

## Discussion

We have shown how a DL model can be developed for assessment of IQ in T2-weighted spin-echo prostate MRI. When IQ scores are further binarized into “nondiagnostic”, defined as requiring a rescan, or “diagnostic”, defined as not requiring a rescan, the model has an accuracy of 78.3% and the AUC of the receiver operating characteristic curve is 0.867.

We further illustrated how a DL model could be used in improving the efficient use of MR scanner time by advising whether a newly reconstructed T2WI scan is nondiagnostic, requiring a rescan, or not. We acknowledge that the rate of false-positive scans was exceptionally large in this illustrative example (73/116, 63%), but we believe this is due in part to technologists wanting to cautiously err on the side of guaranteeing a good quality T2WI series by rescanning. However, even if the false-positive rate were substantially lower, the DL model could eliminate unnecessary rescans. As an example, if ten prostate MRI exams were performed daily on a scanner, and if the number of unnecessary rescans could be reduced by two per day, the savings would be 10 min or more of gradient time, the exact value depending on what sequence is used for rescanning, PROPELLER or possibly a repeat of the original T2WI sequence.

Integration of a model such as this into the clinical workflow has several challenges. A major one is to first gain the trust of the technologist / radiologist system overseers in the accuracy of the model. This is analogous to the need for DL-based PI-RADS interpretation tools, *e.g*. [17], to gain the trust of radiologists if they are to be used in the clinical routine. From a practical standpoint, implementation of T2WI DL quality assessment would ideally be provided as a diagnostic/nondiagnostic advisory signal to the technologist within seconds of completion of the T2WI data acquisition, possibly requiring special-purpose instrumentation to perform the prostate segmentation and IQ labeling quickly.

A byproduct of this work was the evaluation of inter-reader consistency. For the four experienced readers, the mean *κ* for IQ was 0.738, and for nondiagnostic/diagnostic rescanning, it was 0.606, both indicating substantial agreement, comparable to those previously reported, 0.632 [22] and 0.660 [23]. These values can serve as a baseline for the performance of a DL model. In this work, the DL *versus* expert radiologist *κ* for IQ was 0.658, and for nondiagnostic/diagnostic was 0.537.

This work is similar to, but has a few notable differences from, other models. The number of cases included in the training, validation, and testing was somewhat higher, 1,412 in total *versus* 316 [23], 600 [25], or 1,046 [24]. As with previous models, the percentage of uninterpretable exams (IQ = 0) was relatively small compared to other IQ score categories. We attempted to account for this by specifically targeting low-quality images when selecting the data, as well as by implementing minority oversampling in the training set. To attempt to avoid overfitting, augmentation was used whereby the duplicated scans were adjusted by shifting, slight rotations, or left/right reflection. This work also included image sets, which covered a broad range of fields of view and resolution parameters (see Table 1), done to expand the model’s usefulness to a wide variety of acquisitions.

We acknowledge that this model only evaluated one sequence within the mpMRI exam. However, this was done by design, the goal being for the model to advise whether to accept the T2WI result or rescan with PROPELLER while the patient is still undergoing the examination as suggested in PI-QUALv2 [13].

There are several limitations to be taken into account. Despite specific efforts, the percentage of lowest-quality IQ = 0 exams was still relatively small (119/1,412 = 8.4%). To address the class imbalance of a limited number of low-quality training exams, we implemented oversampling of this class in the model training to increase the model’s performance on these scans. Ideally, more true low-quality scans would be added to the dataset. Although the IQ = 0 percentage was low, the percentage of nondiagnostic exams (460/1,412 = 32.5%) was considerably higher, comparable to those reported in other, similar studies, *e.g*., 33.1% [24]. Another limitation is that these datasets were all acquired from 2017 to 2021, many prior to the publication of PI-RADS v2.1. Thus, retuning of models based on more recently acquired datasets would be appropriate.

Another limitation is that only 203 of the 1,412 exams were evaluated by multiple radiologists. Evaluation by multiple readers is expected to improve accuracy and consistency, but reviewing beyond that done here was impractical. We note that the inter-reader *κ* (0.738 ± 0.043) showed substantial agreement, although greater concurrent scoring would give a more accurate evaluation of this number. Importantly, all 203 exams of the test set, against which the DL model was compared, were evaluated by multiple reviewers.

Finally, the study design was retrospective. A prospective study would ideally be designed using data from current protocols, which in our practice virtually never use endorectal coils or 1.5-T field strength in the target patient group.

In conclusion, this study has shown that DL is viable for assessing motion corruption and the overall quality of axial T2WI prostate MRI scans. This assessment could be used to advise in the decision of rescan and thereby decrease the rate of unnecessary rescans in a clinical setting.

## Supplementary information

**Additional file 1:** **Supplemental Table S1.** Counts of the scanner platforms for the acquisitions. **Supplemental Table S2.** Preliminary evaluation metrics for the eleven convolutional neural network models **Supplemental Fig. S1.** Histogram of the distribution of expert radiologist image quality (IQ) scores, after taking the median and breaking ties. **Supplemental Fig. S2.** A visualization of the DenseNet_169 architecture (28). The model consists of four Dense Blocks with a direct connection from each layer to many subsequent layers. Each Transition Layer between Dense Blocks consists of a three-dimensional convolution followed by an average pooling layer of stride 2. **Supplemental Fig. S3.** Two examples of exams having an image quality (IQ) score of 2 or higher (diagnostic) by expert radiologists and model predictions yet were assigned nondiagnostic by the technologist and rescanned using a PROPELLER sequence.

Supplemental Video 1a

Supplemental Video 1b

Supplemental Video 1c

Supplemental Video 1d

## Data Availability

The datasets used and/or analyzed during the current study are available from the corresponding author upon reasonable request.

## Abbreviations

AUC: Area under the receiver operating curve
DL: Deep learning
DWI: Diffusion-weighted imaging
IQ: Image quality
mpMRI: Multiparametric magnetic resonance imaging
MRI: Magnetic resonance imaging
PI-QUAL: Prostate Imaging Quality
PI-RADS: Prostate Imaging-Reporting and Data System
T2WI: T2-weighted imaging
