# Supplementary material for: Deep learning for quality assessment of axial T2-weighted prostate MRI: a tool to reduce unnecessary rescanning
Source: Eur Radiol Exp. 2025 Apr 29;9:44. doi: 10.1186/s41747-025-00584-z (PMC12040773; doi:10.1186/s41747-025-00584-z)
Supplement: Supplementary file 1 — Additional file 1: Supplemental Table S1. Counts of the scanner platforms for the acquisitions. Supplemental Table S2. Preliminary evaluation metrics for the eleven convolutional neural network models Supplemental Fig. S1. Histogram of the distribution of expert radiologist image quality (IQ) scores, after taking the median and breaking ties. Supplemental Fig. S2. A visualization of the DenseNet_169 architecture (28). The model consists of four Dense Blocks with a direct connection from each layer to many subsequent layers. Each Transition Layer between Dense Blocks consists of a three-dimensional convolution followed by an average pooling layer of stride 2. Supplemental Fig. S3. Two examples of exams having an image quality (IQ) score of 2 or higher (diagnostic) by expert radiologists and model predictions yet were assigned nondiagnostic by the technologist and rescanned using a PROPELLER sequence. [file 41747_2025_584_MOESM1_ESM.pdf]

Deep learning for quality assessment of axial T2-weighted prostate MRI: a tool to reduce unnecessary rescanning

ELECTRONIC SUPPLEMENTARY MATERIAL

Supplemental Table S1   Counts of the scanner platforms for the acquisitions

Scanner	Counts
GE Discovery MR750w 3 T	742
Siemens Skyra 3 T	413
Siemens MAGNETOM Vida 3 T	154
GE Optima MR450w 1.5 T	32
GE DISCOVERY MR750 3 T	28
GE SIGNA Premier 3 T	28
GE Signa HDxt 1.5 T	6
Siemens Aera 1.5 T	3
Siemens MAGNETOM Sola 1.5 T	3
GE SIGNA PET/MR 3 T	3

Supplemental Table S2 Preliminary evaluation metrics for the eleven convolutional neural network models

Model	IQ accuracy (%)	Rescan accuracy (%)	IQ Cohen κ	Rescan AUC	F1 score
DenseNet_264	55.7	75.4	0.61	0.854	0.62
DenseNet_201	53.7	72.4	0.57	0.800	0.63
DenseNet_169	52.7	76.4	0.59	0.836	0.64
DenseNet_121	49.8	69.5	0.55	0.772	0.63
EfficientNet_b2	57.1	76.9	0.64	0.819	0.69
EfficientNet_b1	49.8	70.9	0.57	0.782	0.64
EfficientNet_b0	46.3	64.0	0.31	0.748	0.28
SENet_154	51.7	73.4	0.52	0.825	0.69
SEResNet_152	52.2	72.4	0.53	0.804	0.61
SEResNet_101	46.3	71.9	0.43	0.812	0.66
SEResNet_50	43.8	64.5	0.35	0.715	0.45

AUC Area under the receiver operating curve, IQ Image quality.

Supplemental Fig. S1 Histogram of the distribution of expert radiologist image quality (IQ) scores, after taking the median and breaking ties.

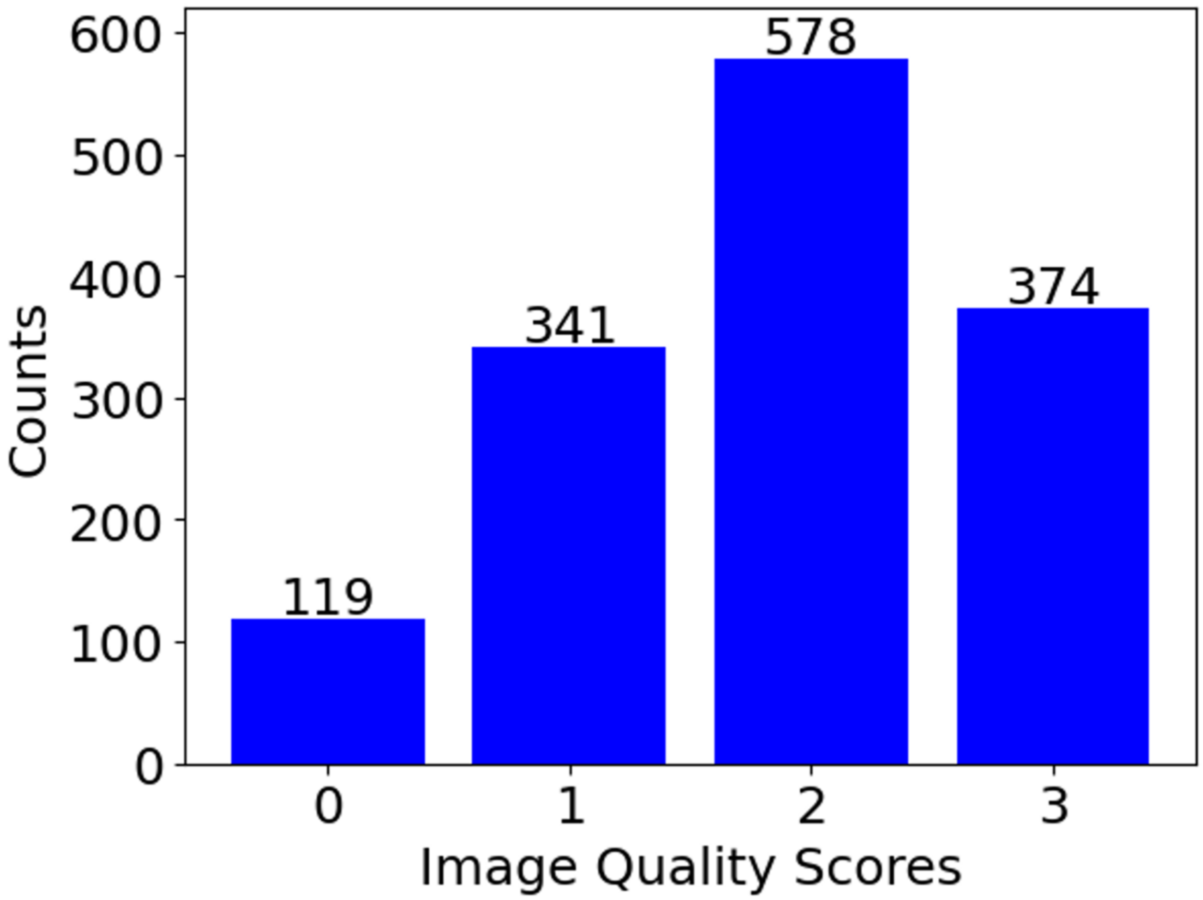

Supplemental Fig. S2   A visualization of the DenseNet_169 architecture (28). The model consists of four Dense Blocks with a direct connection from each layer to many subsequent layers. Each Transition Layer between Dense Blocks consists of a three-dimensional convolution followed by an average pooling layer of stride 2.

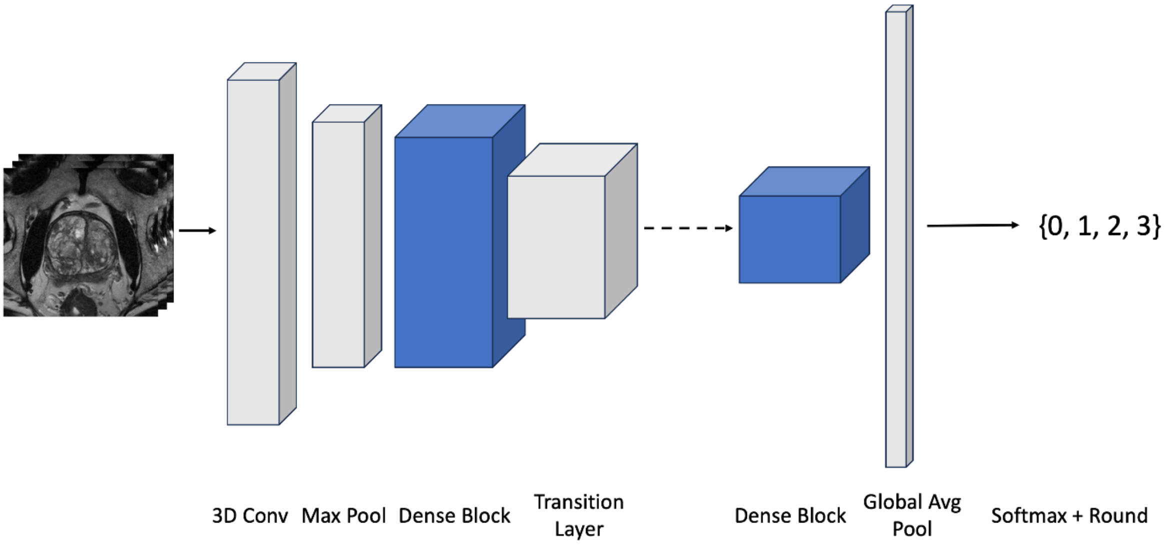

Supplemental Fig. S3   Two examples of exams having an image quality (IQ) score of 2 or higher (diagnostic) by expert radiologists and model predictions yet were assigned nondiagnostic by the technologist and rescanned using a PROPELLER sequence.

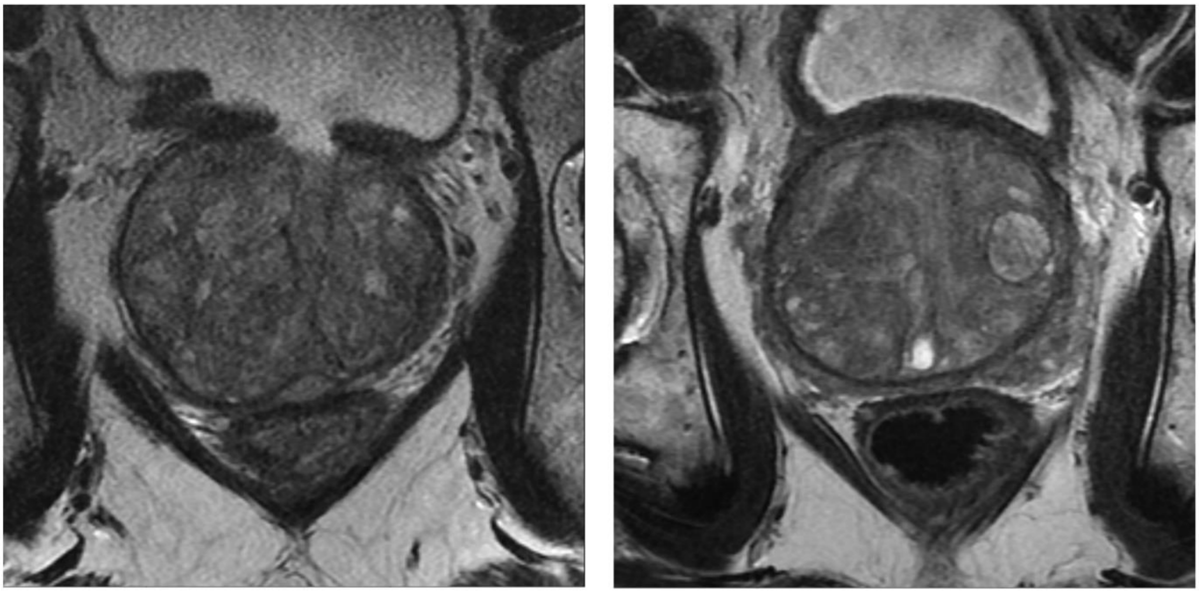

Supplemental Video S1 Scans used as references for scoring: (a) Uninterpretable (IQ = 0); (b) marginally interpretable (IQ = 1); (c) adequately diagnostic (IQ = 2); and (d) more than adequately diagnostic (IQ = 3).
